# Zebras and Biting Flies: Quantitative Analysis of Reflected Light from Zebra Coats in Their Natural Habitat

**DOI:** 10.1371/journal.pone.0154504

**Published:** 2016-05-25

**Authors:** Kenneth H. Britten, Timothy D. Thatcher, Tim Caro

**Affiliations:** 1 Department of Neurobiology, Physiology and Behavior, University of California, Davis, CA 95616, United States of America; 2 Department of Wildlife, Fish and Conservation Biology, University of California, Davis, CA 95616, United States of America; State University of New York Downstate Medical Center, UNITED STATES

## Abstract

Experimental and comparative evidence suggests that the striped coats of zebras deter biting fly attack, but the mechanisms by which flies fail to target black-and-white mammals are still opaque. Two hypotheses have been proposed: stripes might serve either to defeat polarotaxis or to obscure the form of the animal. To test these hypotheses, we systematically photographed free-living plains zebras in Africa. We found that black and white stripes both have moderate polarization signatures with a similar angle, though the degree (magnitude) of polarization in white stripes is lower. When we modeled the visibility of these signals from different distances, we found that polarization differences between stripes are invisible to flies more than 10 m away because they are averaged out by the flies’ low visual resolution. At any distance, however, a positively polarotactic insect would have a distinct signal to guide its visual approach to a zebra because we found that polarization of light reflecting from zebras is higher than from surrounding dry grasses. We also found that the stripes themselves are visible to flies at somewhat greater distances (up to 20 m) than the polarization contrast between stripes. Together, these observations support hypotheses in which zebra stripes defeat visually guided orienting behavior in flies by a mechanism independent of polarotaxis.

## Introduction

Many hypotheses have been proposed for the function of zebras’ extraordinary black and white stripes, including an antipredator defense operating through crypsis [1] or confusion of predators [2], a means of reinforcing social bonds [3], thermoregulation [4], and defense against ectoparasites [5]. Only the last is convincingly supported by experimental [6,7] and comparative evidence [8]. Glossinids (tsetse flies) avoid landing on black-and-white striped objects [5,9,10], and tabanids (horse flies and deer flies) avoid landing on black-and-white striped surfaces, including trays filled with salad oil, boards, balls, and buckets painted with black and white gloss paint [6]. In more natural circumstances, tabanids attack white horses less than dark horses [11]. These and other studies led Horvath and his colleagues to infer that tabanids avoid zebra pelage, although they remained agnostic as to mechanism, since white and black surfaces modulate both luminance and polarized light [6]. They found that white coats of European horses reflect light with a much lower degree of polarization (*d*) than brown or black coats near Brewster’s angle [11] and went on to propose that white coats diffusely backscatter light, thereby both reducing the degree of linear polarization and rotating the angle of polarization of the emitted light. On the other hand, black coats reflect highly polarized light at or near Brewster’s angle. As tabanids [12] and possibly glossinids [13] can detect polarized light, it is reasonable to assume that white stripes disrupt both the degree and angle of an otherwise attractive black pelage polarization signature. Yet there is an alternative hypothesis: contrasting stripes may simply disrupt the body’s outline [14] or break up the form of the host as they were once envisioned to disrupt the outline of the prey for large carnivores [1].

To distinguish between these mechanisms, we investigated the visibility of stripes and their polarized-light signature in plains zebras in their natural habitat in Africa. We observed modest polarization signals from both white and black stripes, which were similar in their angle of polarization. This result provides little support for a role of stripes in defeating polarotaxis, which in turn suggests that the high-contrast stripe pattern itself is more likely to be protective [15]. While stripes do not camouflage the visibility of zebras to large mammalian predators [16], we consider it a likely possibility that th**e**ir pattern confuses the simpler visual systems of insects.

## Methods

**Fig 1:**
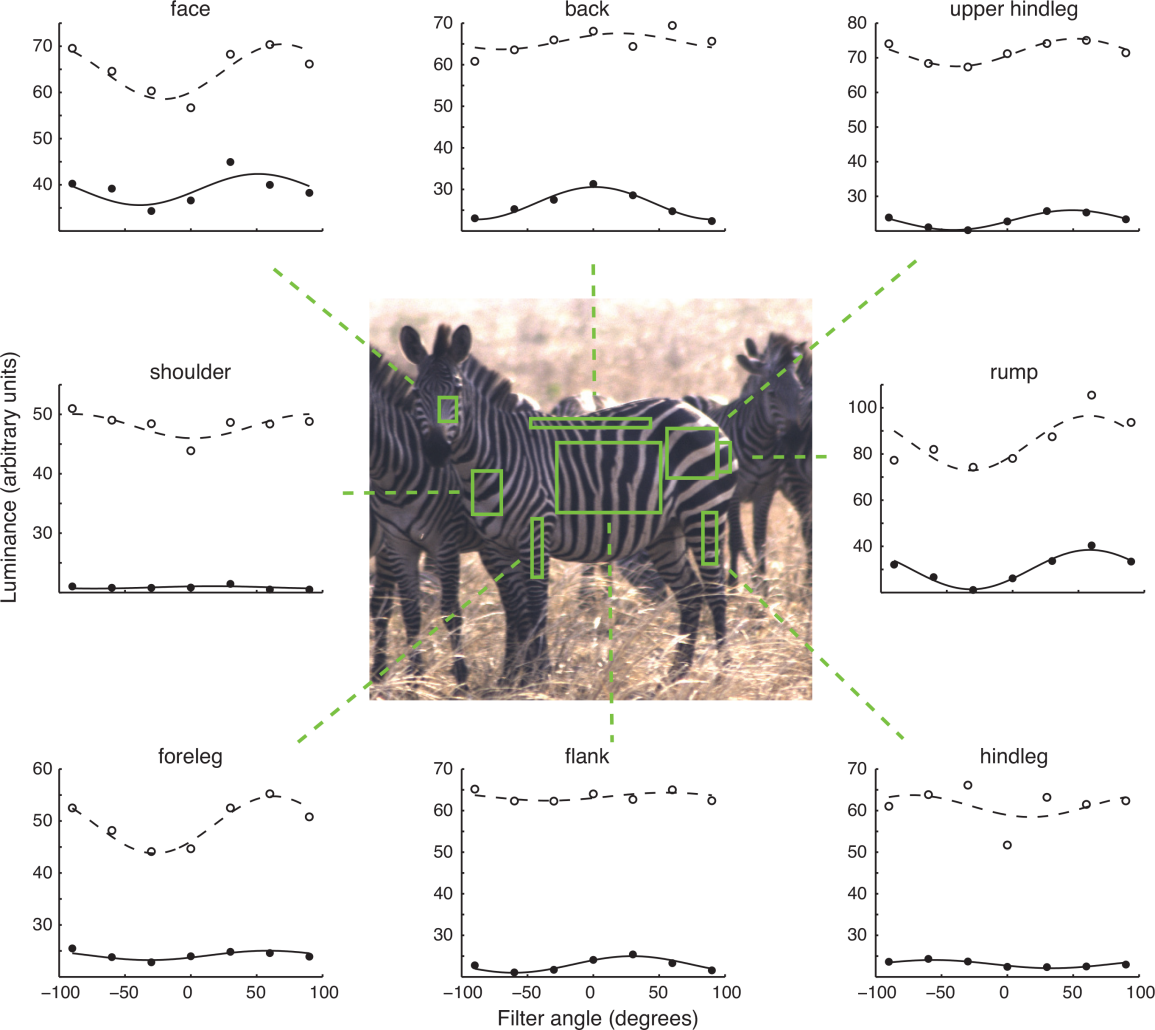
Single zebra data set example. The centre shows a typical zebra image, with eight regions of interest (ROI) denoted by the rectangles outlined in green. These areas were used consistently across individual subjects. For each ROI, the corresponding polarization data are indicated by the surrounding plots. In each, the luminance of the black and white stripes are shown by the closed and open circles, respectively; each is a single measurement from the ROI at that polarization angle. In each series, the -90° and +90° points are replicate, independent observations; they provide an estimate of the repeat reliability of the data. As expected, reliability is greater for the larger ROIs. Superimposed on each data set is the best-fit sinusoidal function (also see Fig 2). The zebra image in the centre corresponds to the individual data points at +30°. The colour scale in the zebra image has been adjusted here for illustration purposes only, because the 14-bit data appear very dark in standard rendering. Note the considerable change in luminance through filter angles in white stripes in most ROIs.

To examine the degree of polarization and plane of polarization of reflected light from both black and white stripes on coats of zebras, we took images of 21individual plains zebras (*Equus burchelli*) in Katavi National Park, Tanzania. All necessary permits were obtained from the Tanzania Wildlife Research Institute, Tanzania Commission for Science and Technology. UC Davis IACUC approval was not required because neither the animals nor their habitat was disturbed in any way. The study site was located at latitude 6°35’–7°05’S, longitude 30°45’–31°25’E. We took photos in the dry season in July through mid-September 2012 at a distance of 35–101 m as determined by rangefinder. Local shading, scene haze, sun azimuth and elevation were noted for each subject; in general there was little heat distortion and little overall scene shade. The camera was a Nikon D50 with a 100–300 mm Nikkor lens fitted with a linear/circular polarizing filter designed for digital cameras (Digital Quantaray Professional Filter Series 62 mm Circular PL). The majority of our images were taken with the viewing angle within +/- 45 degrees of the body axis of the zebra (see S1 Table.) Images were captured using either 270 (1 series) or 300 mm focal distance (20 series), and the camera/lens was empirically calibrated with respect to image magnification by photographing objects of known size at a known distance. For the 300 mm setting, the image resolution was 89.5 pixels/degree. For each individual animal, a series of 7 images were taken in very quick succession at a time when subjects were relatively sedentary and were not jolting their heads or being bothered by flies. The hand-held camera lens rested on a half-opened car window with the polarizing filter manually rotated through 180° at 30° intervals, starting and ending with the filter axis (minimum transmission) vertically oriented. Invariably, the camera exposure was controlled manually and was not changed within each series. The majority of the images were obtained at a single site, a water hole surrounded by grassy vegetation. Up to 8 individual regions of interest (ROIs) were localized (Fig 1), and tracked through the image series. The quantitative results presented here were derived from a set of 21 individuals from which sufficient data were obtained (average of 6.9 ROIs per zebra). Our criteria for including a ROI were that it be visible through the entire series of images and not be occluded by other body parts or by vegetation; one ROI was excluded because of luminance artifacts. We did not attempt to isolate nearly planar regions or to control the angle of the surface relative to the illumination or the direction of view, and the azimuthal angle of the zebra’s body axis ranged through 360°. The biting flies of interest forage at approximately 1 m above the ground, and are typically most active in the middle of the day, the time when most photographs were taken, so our images would probably be representative of what flies would view when approaching zebras from downwind. Some of our ROIs were flanking regions but some were of the back and rump (see Fig 1) that were not perpendicular to our line of sight as viewed through the camera or by an incoming biting fly. Such oblique surfaces, if illuminated by high-elevation midday sunlight, could provide directly reflected light with incident and reflectance angles near 45°. Because of the complex geometrical structure of hair, and because the surface orientations are impossible to estimate from 2D images, the actual light reflectance angles are statistical at best and not available for analysis from field data.

Images were stored in 14 bit/channel raw NEF files (RGB format) and converted to 48-bit (16 bits/channel) TIFF files using the public domain software ‘dcraw’ (v. 9.16, Dave Coffin), using a setting that conserved the sensor values for each pixel. All subsequent analysis was performed in Matlab (Mathworks, Inc.). These RGB images were converted into grayscale using Matlab function *rgb2gray*, which scales the raw sensor values to CIE luminance. Note, however, because the camera was not calibrated using a known luminance source, the resulting values are linearly related to luminance by an unknown scale factor. This scale factor depends on the exposure setting for that series; however, exposure settings were never changed within a series, although they varied considerably across series.

ROIs were identified and matched by hand across images using fiduciary marks on the zebra’s coat (Fig 1). The black and white stripes in each individual image ROI were segmented using an adaptive thresholding algorithm (Matlab function *graythresh*) that maximized luminance variance between black and white stripes relative to within-stripe variance. This algorithm reliably identified the same stripes in each of the subject’s 7 images, even if their luminances changed because of polarization effects or animal movements. By virtue of the feature tracking and adaptive thresholding, our approach is less sensitive to movement artifacts than is imaging polarimetry.

**Fig 2:**
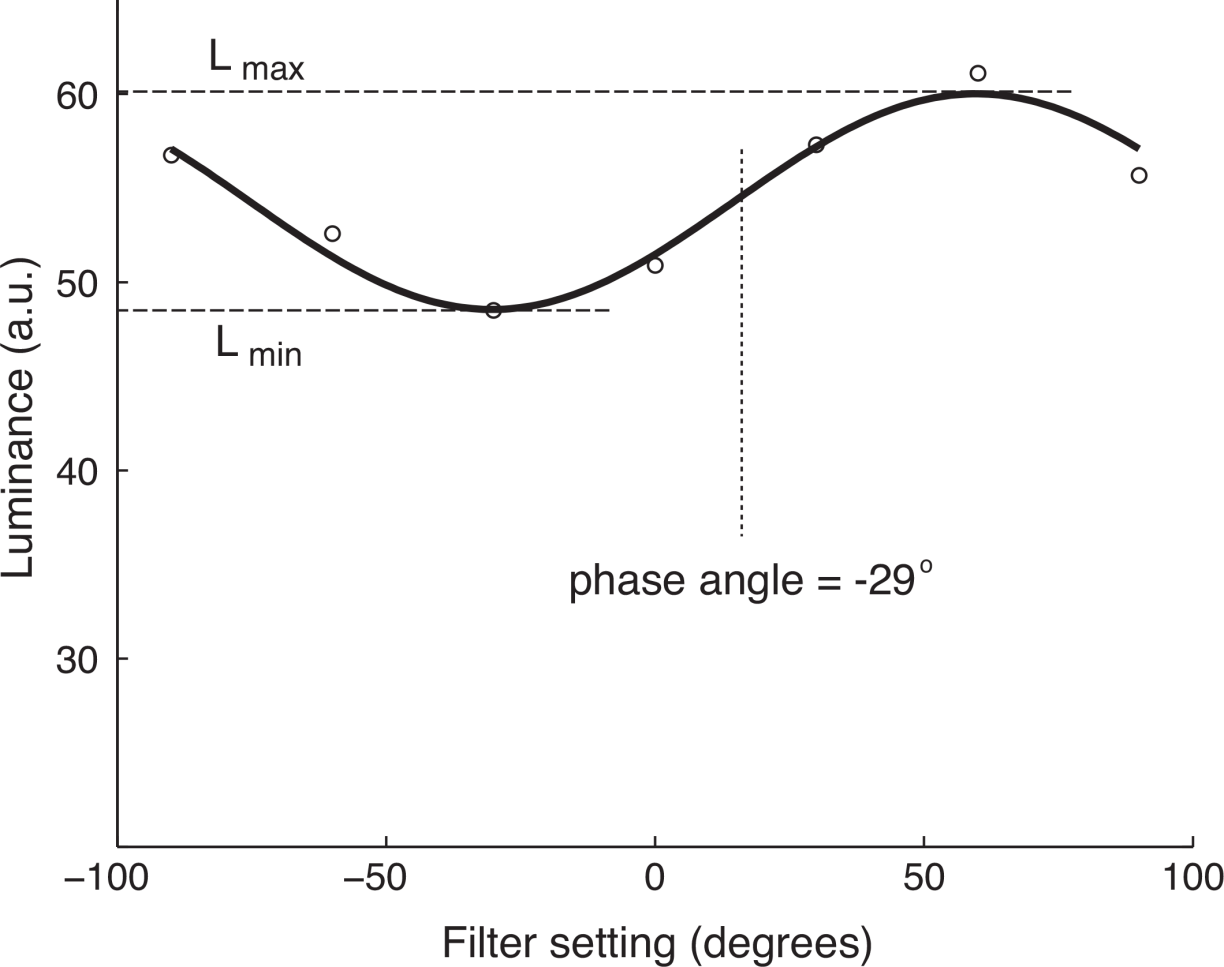
Derivation of extracted quantities from fitted sinusoids. This shows a single fit to the white stripes from a single ROI (foreleg in Fig 1). The phase angle corresponds to the offset of the fit from odd symmetry around the midpoint filter setting, which in this case was approximately 30 degrees. The *d* value is calculated from the contrast between the maximum and minimum values of the fit curve. It is important to note that the phase angle parameter is not the same as the angle in which more polarized light is present, but is linearly related to this physical parameter. See methods for details.

To determine degree and angle of polarized light reflected or scattered from each region, we fitted the luminance by a 180° period sine function of the polarization filter setting (Fig 2), according to the expression

L=a×sin(2θ+ϕ)+b (1)

where Θ is the polarization filter angle, ranging from -90° to +90°, and Φ is the phase of the best-fitting sinusoid, and a and b are free parameters for the amplitude of the function and the baseline luminance. With our filter angle convention, a phase angle of -45° corresponds to a fit function with luminance peaks with the filter set at +/- 90°, which means the *minimum transmission angle* is vertical, and thus indicates a preponderance of horizontally polarized light. The orthogonal phase angle of +45° indicates a distribution of polarization angles biased in favour of vertically polarized light. Through the rest of the paper, we will refer to the quantity extracted from our fits as the phase, and refer to the physical properties of the light in the images as “polarization angle”. We chose our angle conventions so that the range of greatest interest (horizontal to vertically polarized light) would appear in the middle of the range of phases in figures. The sinusoidal fits to our data were quite good; average correlations between fit and data were 0.73 and 0.66 for black and white stripes, respectively. Because the phase parameter of the fit is unreliable for images with low *d* values, we restricted our quantitative analysis of phase angle to regions where there was a significant correlation between the data and the fit (r > 0.71, df = 6, p < 0.05) for both the white and black stripes in the ROI.

The maximum and minimum values of this best-fit function were used to calculate the degree of polarization, *d*, using the expression

$$d=(L_{\max}-L_{\min})/(L_{\max}+L_{\min})$$ (2)

where L_max_ and L_min_ refer to maximum and minimum luminances, respectively (Fig 2). This parameter captures the fraction of emitted light that is concentrated in a particular angle of polarization. A value of 1.0 means that 100% of the reflected light is polarized in a single plane, while a value of 0 results from light containing equal amounts of all polarization angles (as occurs for reflections from matte surfaces).

Michelson contrast between black and white stripes was calculated similarly

$$contrast=(L_{white}-L_{black})/(L_{white}+L_{black})$$ (3)

where L_white_ and L_black_ denote the average luminance of the white and black regions, respectively. Michelson contrasts were averaged across polarization filter settings to produce a single value for each ROI.

For the analysis of polarization and contrast against distance, we filtered the original luminance images using a Gaussian blur function with a width of 1°. As we knew the distance to each zebra by rangefinder, we could scale the filter to simulate the blurring effect of viewing from a variety of distances. Original ROIs and stripe segmentation boundaries were used for the analysis of blurred images, but the filter was applied to the entire image to minimize boundary effects.

We looked at background polarization signals by extracting from the same images paired background regions, located adjacent to individual ROIs in our primary analysis. These were usually patches of dry grass that were invariably still and not blowing in the wind. Note that plains zebras spend much of the dry season inhabiting plains [17]. We could then compare the average *d* value from the zebra to the *d* value of the light reflecting off the background vegetation.

## Results

### Degree of polarization

**Fig 3:**
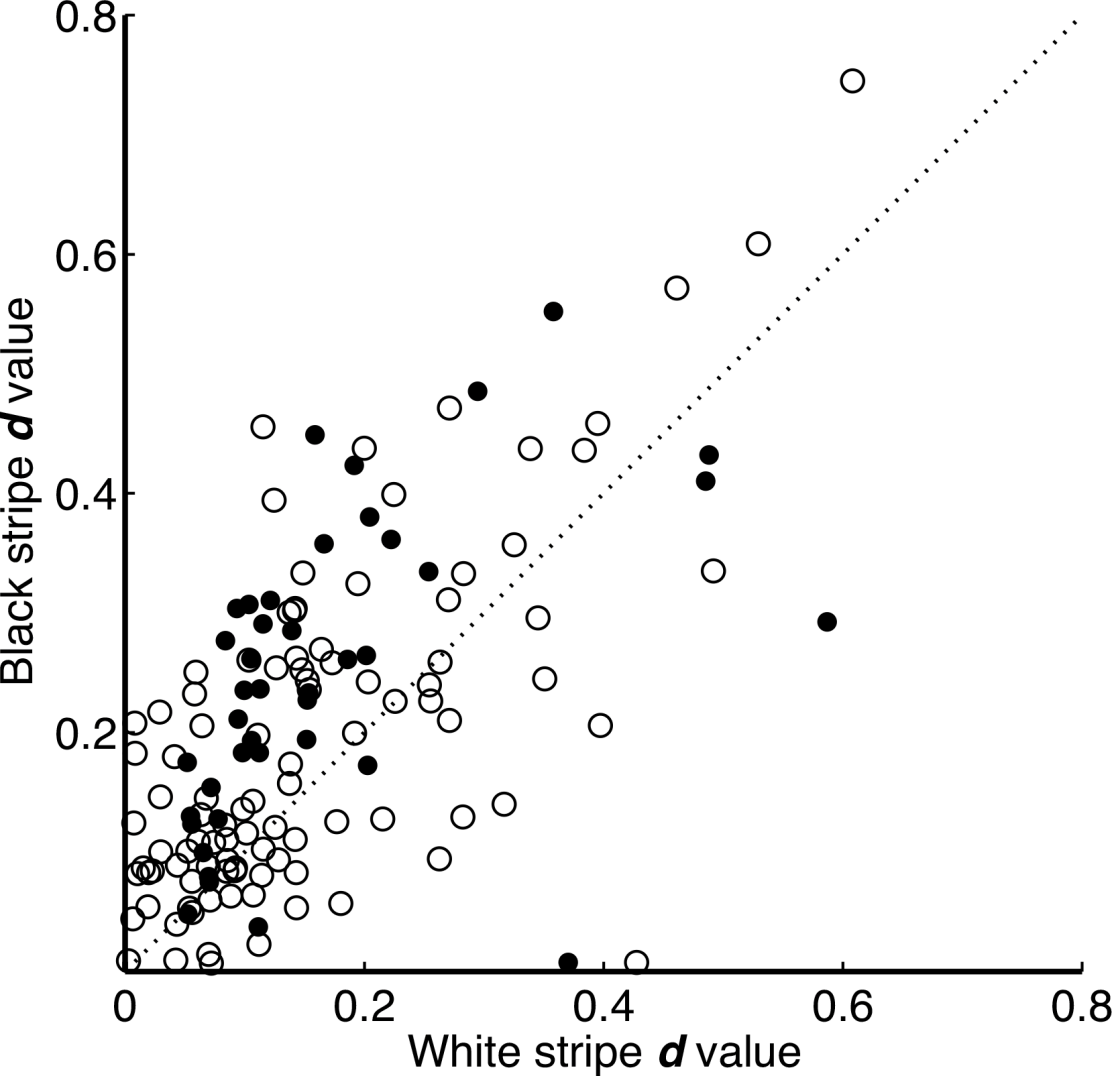
Degree of polarization signals in black and white stripes. The filled circles represent the subset of ROIs used in Fig 5, where there was a significant correlation between fit and data; these are the same cases illustrated in Fig 5.

Both black and white stripes reflect substantial amounts of polarized light, and the degree of polarization was highly correlated between the black and white stripes in each ROI (r = 0.63, df = 139, p < 10^−16^; Fig 3). Thus while there was a considerable range of *d* values across zebras and ROIs, the black and white stripes within each region were similar in their degree of polarization. Nonetheless, black stripes had a greater degree of polarization, with a median difference of 46% (Wilcoxon signed rank test signed rank = 2286, n = 141, p < 10^−7^). Because each zebra was sampled multiple times, we verified that the difference between stripes was genuinely significant using ANOVA, in which stripe was a fixed effect and zebra was treated as a random factor (p = 0.003; see S2 Table).

**Fig 4:**
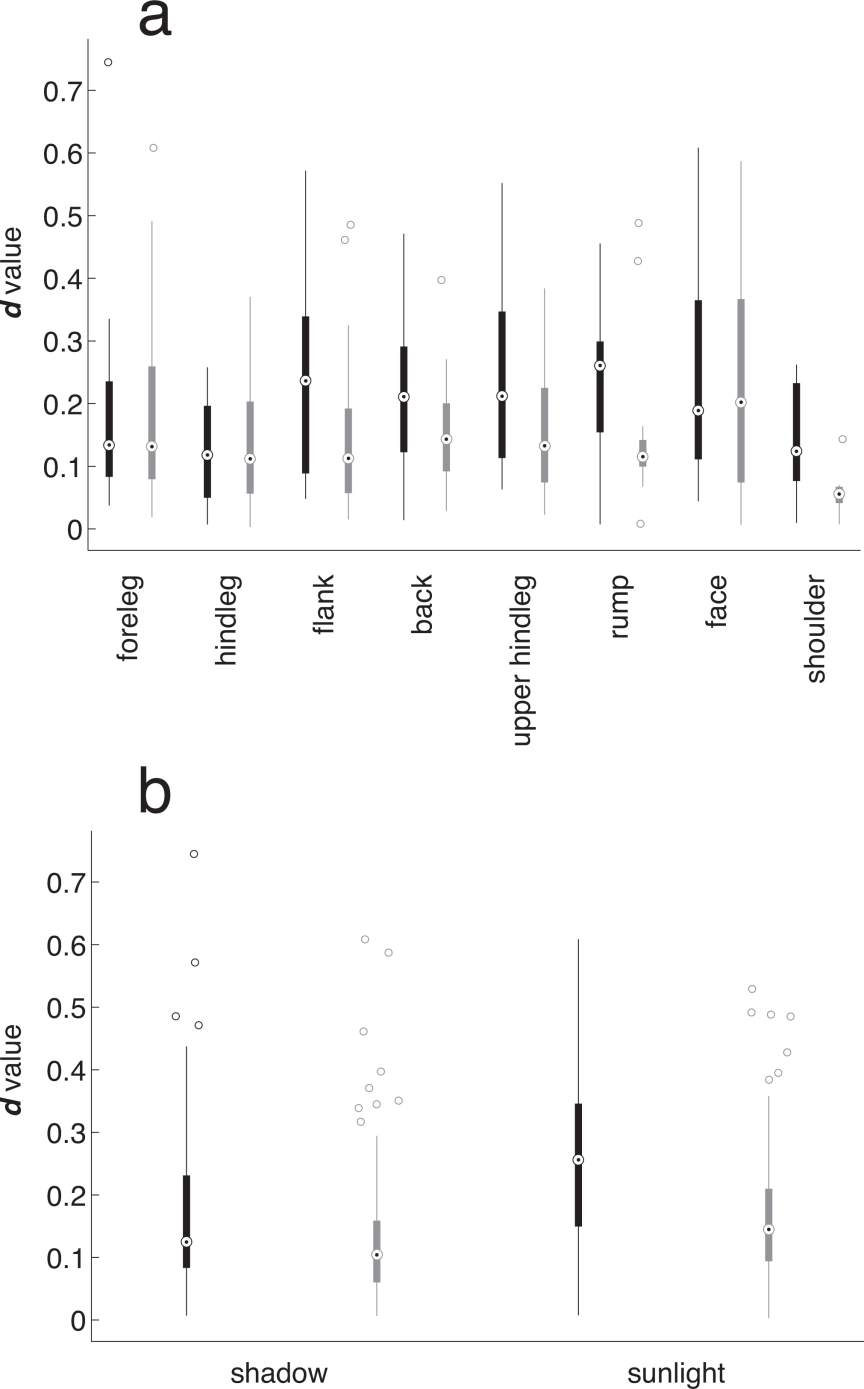
Box/whisker plot showing the distributions of *d* values in different ROIs for black and white stripes under different illumination conditions. In each column, the dot surrounded by a circle depicts the mean, the box shows the quartiles, the whiskers the 95% confidence intervals, and the “o” symbols show outliers.

We were interested in what factors would influence the degree of polarization, and the difference between black and white stripe *d* values. Firstly, we performed a multi-factor ANOVA, testing variables that would be expected to have an influence: the ROI and the presence or absence of sunlight. The most consistent effect was due to zebra identity (treated as a random factor), which influenced both black- and white-stripe *d* value (ANOVA, p values < 0.03; see S3 Table). Black-stripe *d* values were influenced by whether they were in direct sunlight (p = 0.033), but this was the only direct illumination effect. White stripes were not affected by either region or illumination. To graphically illustrate these effects, Fig 4 shows (a) the range of *d* values for black and white stripes from different ROIs, and (b) when ROIs were directly or indirectly illuminated. The regions that systematically showed the greatest differences between black and white stripe *d* values were the shoulder, flank, back, upper hindleg, and rump. Also, black stripes consistently had a higher *d* value than white stripes, and sunlit regions consistently had a higher *d* value than shaded regions, both of which one would expect from optical principles. However, none of these differences were substantial, as is evident from S3 Table and Fig 4.

In the preceding analysis, a large number of scene variables could have been included in the effect of zebra identity, such as sun azimuth, elevation or the orientation of the zebra. Because each zebra was only sampled once, it is difficult to differentiate between illumination conditions and intrinsic differences in the pelts of the animals, such as amount of dust or oils in the hair. However, we could explore systematic effects of viewing conditions by ignoring zebra identity in analyzing our image data. We therefore performed an ANCOVA analysis in which we examined the effects of sun azimuth (east or west), sun elevation (low or high), body region (1–8), and animal orientation (continuous). We also included the one previously significant predictor: illumination on the ROI. No additional effects were revealed in this analysis; the only effect was of direct illumination, and only on black stripes (p = 0.016; S4 Table). This analysis suggests that the significant differences between individuals in our data set resulted from intrinsic differences in their pelage, rather than from the circumstances under which each was photographed.

Although not significant in the preceding analysis, the mean *d* value for high-elevation sun was 27% higher than for low-elevation sun. We might expect this difference to be most pronounced for dorsal regions viewed in low-angle sunlight from behind, given previous work [6]. Five samples of dorsal ROIs met these criteria, and in these cases, the mean black-stripe *d* value was 38% higher than in all other ROIs, though this difference was not statistically significant (t = 1.14, df = 139, p = 0.26). Interestingly, the white-stripe *d* values are also 17% higher in these regions, consistent with there being a substantial surface reflection signal from white stripes as well as black.

### Angle of polarization

**Fig 5:**
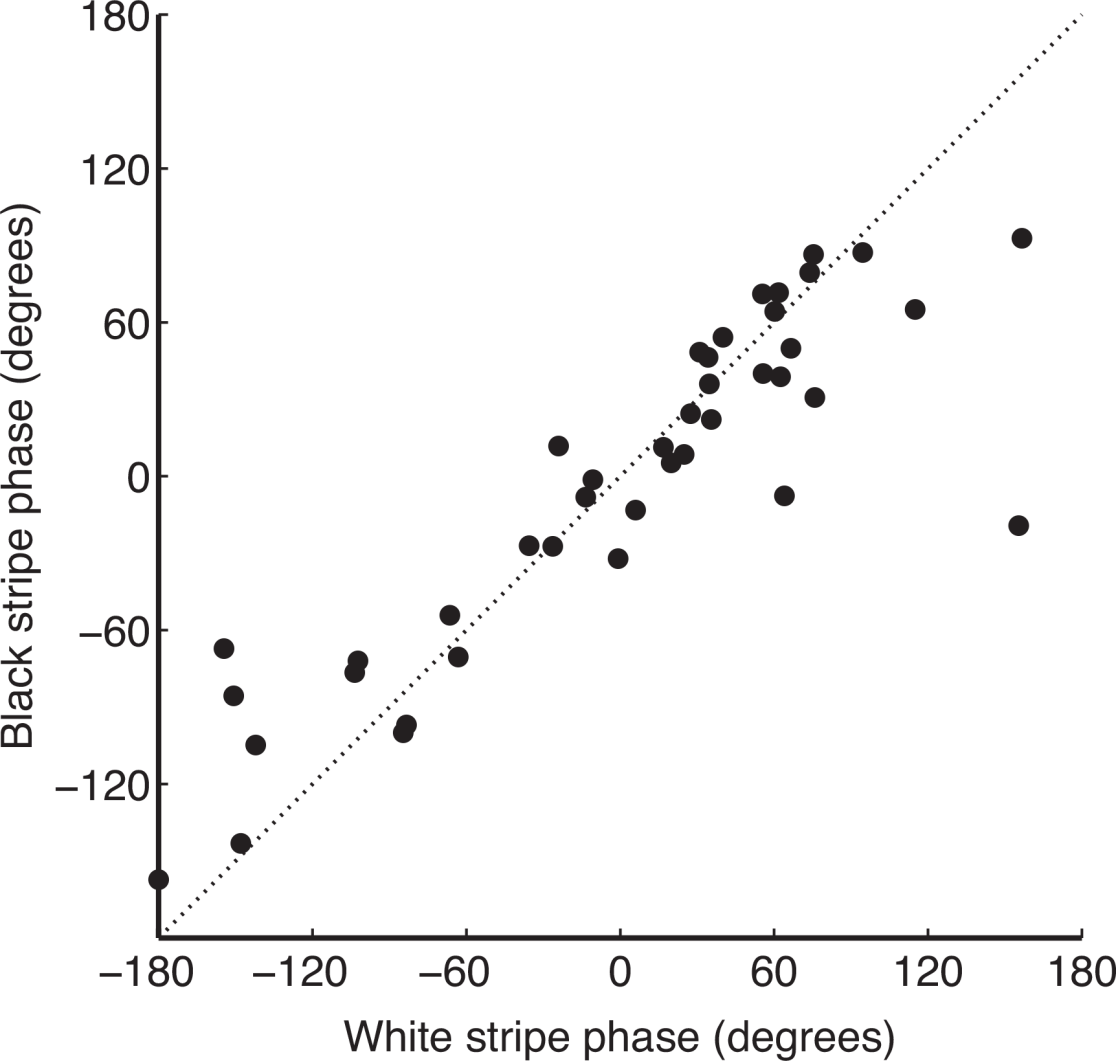
Correlation between fit phases in black and white stripes. Each data point represents a single ROI in a single zebra, and the phases of each were fit independently. Note the high degree of correlation evident in the data; outliers were present but rare. Because phase could only be accurately estimated in data where the *d* value was high, this analysis is restricted to the cases where there was a significant correlation between data and fit.

The effect of polarized light on the visual system of a fly approaching from a distance might be influenced not only by the magnitude of the polarization signal, but also by the relative polarization angles in the two sets of stripes. If the angle of polarization of emitted light were systematically orthogonal between adjacent black and white stripes, then the striping might actively counteract the signal from the black stripes. Therefore we also analyzed the angle of polarization of the light from our images. The angle of polarization was evident in the phase of the fit of the sinusoid (Fig 2; see methods). Forty of our ROIs met the criterion of a significant correlation between data and fit, and the correlation between their phase angles is shown in Fig 5. Black and white stripes showed similar phases, and were highly significantly correlated (r = 0.89, df = 40, p < 10^−13^). The majority of cases had polarization angles within 45°, and there was no significant difference between black and white phases (paired t test, t = -0.66, p = 0.51, df = 41) Therefore, we conclude the white stripes cannot counteract the polarized-light signal from the black stripes, because of the consistency of the phases of the emitted light from both.

### Effects of distance

**Fig 6:**
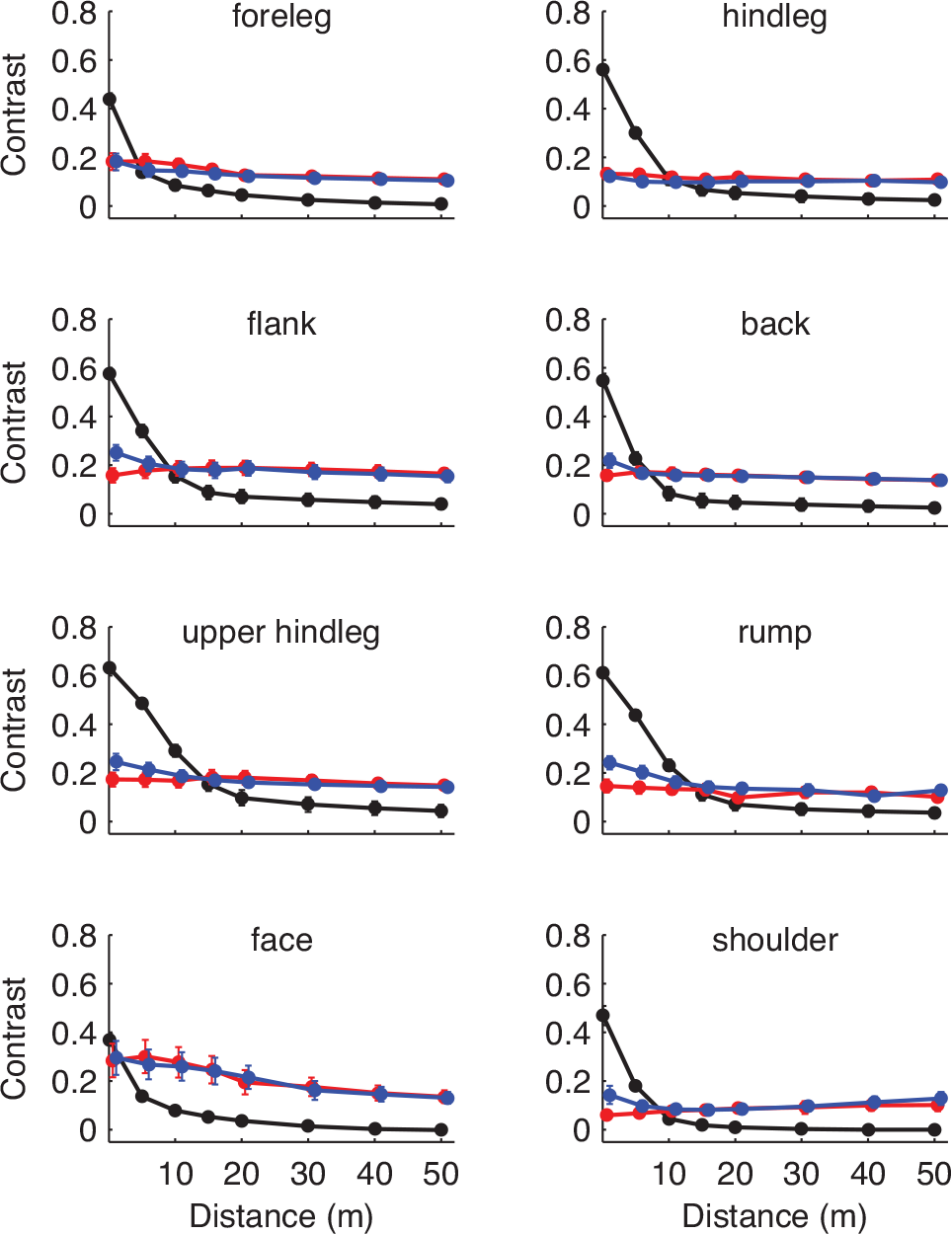
Visibility of polarization and stripe contrast with respect to distance from the zebra, plotting each ROI separately, averaged across individuals. The Y axis represents contrast units, which are *d* values for the polarization data (blue denotes black stripes, red denotes white stripes), and Michelson contrasts (black lines) for the luminance data. Both are equivalent, unitless measures of the corresponding optical quantity. Error bars depict S.E.M., and where they are not visible, they are smaller than the plotting symbol.

When parasitic flies are tracking hosts, they are thought to shift from olfactory homing to visual homing at relatively short distances [18] and we were interested in estimating the distances at which different features of the zebra would be visible to a fly tracking upwind. Tabanids respond visually to host animals at a distance of about 15 m [19], and glossinids abruptly turn towards a visual target from within an odour plume when the target is presented at a distance of 3 m [20]. This behaviour is probably a consequence of the low acuity imposed by the limitations of compound eyes. While there are no good estimates of the acuity of the endemic tabanids at our study site, we suspect they are similar to the well-studied blowfly, *Calliphora*, which has a visual acuity of approximately 1° [21,22]. To examine the visibility of zebra features as a function of distance, we filtered each of our images to emulate a 1° Gaussian blur function at different distances from the zebra. For each of these filtered images, we analyzed the same ROIs that were used in the primary analysis. We calculated the *d* value for the black and white stripes, as well as the luminance contrast between the black and white stripes at several distances out to 50 m (Fig 6).

Two important results emerge from this analysis. As expected, the Michelson contrast drops steeply with distance, and depending on the size of the stripes, the stripes become invisible at moderate distances of 5–20 m. Also, the distinction between the two *d* values drops at a similar distance, for the same optical reason. But because the *d* values of the two stripes were similar to begin with (Fig 3), they converge to a nonzero value. Thus at moderate to large distances beyond 20 m, a polarization signal from the whole animal is easier to see than the stripes themselves. Again, this result suggests that stripes are a poor mechanism to defeat polarotaxis, though only if the *d* value of the zebra coat is higher than that of the background.

To address the question of the relative polarization between zebra and background, we analyzed 10 images where we found a nearby patch of grass adjacent to one of our ROIs in the same illumination. We found that the zebra pelts had a much higher *d* value (average of black and white stripe values) than the surrounding vegetation (median ratio 2.06), and this difference was significant (paired t = 2.54, df = 9, p = 0.032). Therefore zebras present a distinctive polarization signal, averaged across the black and white stripes, which would be available to a fly approaching upwind and crucially, at distances where the stripes themselves would be invisible to the fly.

## Discussion

Three main findings emerge from this study. First, substantial polarized light reflectance signals are seen from both black and white striped pelage of free-living zebras. Second, these signals are polarized in a similar plane. Third, the polarization of light reflecting from zebras is significantly higher than from surrounding vegetation, so that a positively polarotactic insect would have a distinct signal to guide a visual approach to the zebra. Consequently, it is highly improbable that the primary function of zebra stripes is to defeat polarotaxis by biting flies.

The results from this work are superficially similar to related work on temperate zone ungulates, but different enough to challenge the polarotaxis hypothesis [12,23]. We agree that polarization signals are greater on dark surfaces than on white, and are greater in sunlight than in shade. Indeed, these effects are nearly inevitable from optical principles [24]. The differences between our research and previous work conducted by Horvath’s group–especially the polarization differences between black and white regions—cannot be attributed to the camera being set at an angle perpendicular to the zebra’s flank, as many of our ROI’s (such as the rump and back) were oblique to this angle (Fig 4). Instead we believe there are other explanations for why the difference in the degree of polarization between black and white stripes is modest in the wild, and why the angle of polarization is consistent between the two sorts of stripes. The most striking *d* values from the Horvath work on live animals were found on dark, dorsal surfaces illuminated by low-angle sun. In the tropics, such illumination conditions are less common, and would mostly occur in the early morning or late afternoon, when tabanids are less actively foraging [25,26]. However, our data included a few observations under such illumination conditions, and we observed neither strikingly high black-stripe *d* values nor strikingly greater differences between black and white stripes across ROIs (Fig 4). This suggests that there are differences between the pelage of zebras in their natural habitat and that of temperate zone ungulates. The pelage of zebras is certainly very thin compared to other artiodactyls [8], and likely thinner than temperate zone domestic horses, a factor that would probably increase the relative *d* values from the white zebra stripes. Thicker pelage would contain larger number of hairs that light could interact with, which would increase the length and complexity of light paths and thus provide more opportunity for Rayleigh scattering or reflection from randomly oriented surfaces. By contrast, the thin pelage of zebras would encourage a greater fraction of light to be reflected in a systematic way, and thus carry a stronger polarization signal. In detailed measurements of the optical properties of orderly, light-coloured human hair, light reflects from both the front and back surfaces of hair fibres, and the polarization angle is similar for both and similar to that from dark hair [27,28].

One observation from Horvath’s body of work seems most comparable to ours: imaging polarimetry of a preserved zebra pelt [6]. Unfortunately, this observation is qualitative, so it cannot be directly compared against our data. Museum pelts are often treated to maintain the gloss of the hair, and this might explain the apparently higher *d* values in those images. Certainly, the specimen would have less dust than wild zebras, and dust on the hair would both reduce overall polarization and also reduce differences between black and white stripes. Free-living zebras do roll in dust, as witnessed in the Katavi zebra population, and dust is always in the air during the dry season in East Africa when our photographs were taken.

Our data provide a challenge to the polarotaxis hypothesis for the adaptive significance of zebra stripes for three reasons. Polarization signals from the white stripes of free-living wild zebra pelts are substantial and highly correlated with those of adjacent black stripes, both in terms of degree (magnitude) and polarization angle. At the range where flies are thought to transition from olfactory to visual homing (5–10 m), the optics of tabanid eyes would substantially blur the signals of black and white stripes (Fig 6). The resulting signal appears distinct from surrounding dry grass, at least in our study site, and would thus still be available to attract polarotactic insects. If defeating polarotaxis is not the principal adaptive advantage of stripes, we need to consider alternative mechanisms for why biting flies manifestly avoid striped surfaces [5,6,9,10].

Stripes were once hypothesized to conceal prey outlines from mammalian predators such as lions (*Panthera leo*) or spotted hyaenas (*Crocuta crocuta*). Originally, Thayer [1] suggested that stripes disrupt the outline of the body [see also 14]. He believed that some stripes but not others might blend in with the background, and that internal contrasts would attract the viewer’s attention; both effects would lead the eye away from the outline of the prey, causing the predator not to notice it. Given that zebra stripes are difficult to resolve for large predators unless viewed at very close range [16] and that zebras are a favoured prey of lions [29], and that striping intensity shows poor geographic congruence with large predators in Africa [4,8,30], the antipredator hypothesis of zebra stripes is now untenable.

However, the same logic might apply better to the simpler visual systems of insects. Dipterans have well-studied visual reflexes, two of which are likely to contribute to the host-orienting behaviour of tabanids and glossinids. Many insect taxa will “fixate” to high-contrast objects, orienting either walking or flying behaviour towards the stimulus (mosquitoes [31]; houseflies [32]; *Drosophila* [33]). We have shown that the stripes themselves become indistinct at the moderate distances where tabanids visually orient; therefore the flies must orient to the overall outline of the animal. White stripes could reduce the luminance contrast between the zebra and the background vegetation, compared with a uniformly dark animal, and thus reduce the salience of the zebra as a stimulus for orientation. Our data do not allow us to test this idea directly, since we studied only a single background type. Luminance contrast is in general a poor image-segmentation cue, because of the large effects of partial shading, and chromatic contrast is more reliable. Tabanid color vision [34] could be used in approaching their hosts, but more behavioural data are needed.

Another possibility is that the stripes disturb optomotor responses for approach or landing manoeuvres. Looming stimuli provoke landing responses if they are in frontal vision, but avoidance turns if presented to the side of a fly (for review, see [35]). Patterned stimuli provoke fewer landings relative to uniformly dark ones (glossinids [36]; *Drosophila* [37]). One appealing suggestion for the mechanism for these effects comes from recent theoretical work by How and Zanker, who demonstrate that high-contrast stripes can produce erroneous motion signals [38]. Such errors might contribute to failed landings, which likely depend on accurate motion analysis (*e*.*g*., [39]). The two hypotheses of concealment from flies and confusion of flies are not exclusive; they operate at different distances. We speculate that the stripes might conceal the zebra at moderate distances from flies, and disturb fly landing behaviour at close range. Direct test of these ideas will require controlled behavioural study in the laboratory.

## Supporting Information

S1 Table Summary statistics of the images that were analyzed for this paper. Sun azimuth is relative to true north, and elevation is relative to the horizon, as usual. Body angle was estimated by eye, and is relative to the viewing direction, with 0 degrees indicating a perpendicular view with nose right and 180 degrees perpendicular with nose left.
(PDF)

S2 Table ANOVA relating *d* value to stripe identity and zebra, which was treated as a random factor. (PDF)

S3 Table ANOVA relating degree of polarization to region illumination, region, and zebra. Region refers to which area of the body was covered by each ROI, sun refers to whether an individual ROI in a given series was directly or indirectly illuminated, and zebra (random factor) refers to the individual out of the 21 series that comprised the data set. Together, these variables accounted for 42% and 32% of the variance in *d* values in black stripes and white stripes, respectively. This and the analysis presented in S3 Table were performed using function ‘anovan’ in Matlab, using Type III sums of squares.
(PDF)

S4 Table ANCOVA relating degree of polarization to a set of scene variables that might be expected to contribute to the differences between zebras seen in the analysis reported in S2 Table. Sun azimuth was coded either east or west, because the data were taken in the tropics and the range of azimuths was therefore limited. Elevation was coded as low or high, with the criterion elevation being 45 degrees. The sun variable was as in Table 2, and orientation was a continuous variable, measured in degrees relative to the viewing direction, with 0 being head-right and 90 being head-away. Only illumination was individually significant, though many variables approached signficance. These independent variables accounted for 21% and 13% of the variance in black- and white-stripe *d* values, respectively. We interpret these results to mean that scene variables contributed to the differences between zebras, but that there was considerable residual variance that must be attributed to intrinsic differences between the reflectance properties of the pelts of different zebras.
(PDF)
