# Supplementary material for: Zebras and Biting Flies: Quantitative Analysis of Reflected Light from Zebra Coats in Their Natural Habitat
Source: PLoS One. 2016 May 25;11(5):e0154504. doi: 10.1371/journal.pone.0154504 (PMC4880349; doi:10.1371/journal.pone.0154504)
Supplement: S1 Table — Sun azimuth is relative to true north, and elevation is relative to the horizon, as usual. Body angle was estimated by eye, and is relative to the viewing direction, with 0 degrees indicating a perpendicular view with nose right and 180 degrees perpendicular with nose left. (PDF) [file pone.0154504.s001.pdf]

zebra ID	sun azimuth	sun elevation	body angle	no. of regions
1	70	9	180	6
2	313	53	225	3
3	300	37	180	7
4	299	38	180	8
5	314	54	0	6
6	355	64	0	7
8	70	14	0	5
9	43	56	180	7
10	73	2	0	6
11	62	13	0	6
12	317	59	180	7
13	292	27	45	7
14	74	3	180	7
15	56	50	315	6
16	355	68	45	8
17	349	67	180	8
18	297	41	45	8
19	289	19	0	7
20	31	65	180	8
21	285	25	180	7
22	78	30	0	6

Table S1. Summary statistics of the images that were analyzed for this paper. Sun azimuth is relative to true north, and elevation is relative to the horizon, as usual. Body angle was estimated by eye, and is relative to the viewing direction, with 0 degrees indicating a perpendicular view with nose right and 180 degrees perpendicular with nose left.
