# Supplementary material for: Zebras and Biting Flies: Quantitative Analysis of Reflected Light from Zebra Coats in Their Natural Habitat
Source: PLoS One. 2016 May 25;11(5):e0154504. doi: 10.1371/journal.pone.0154504 (PMC4880349; doi:10.1371/journal.pone.0154504)
Supplement: S2 Table — (PDF) [file pone.0154504.s002.pdf]

Source	Sum Sq.	d.f.	Mean Sq.	F	Prob>F
stripe	0.1661	1	0.1661	18.06	0.0003
zebra	1.17921	20	0.05896	6.61	0
stripe*zebra	0.17842	20	0.00892	0.59	0.9183
Error	3.60269	238	0.01514		
Total	5.13465	279			

Table S2. Anova relating *d* value to stripe identity and zebra, which was treated as a random factor.
