# Supplementary material for: Zebras and Biting Flies: Quantitative Analysis of Reflected Light from Zebra Coats in Their Natural Habitat
Source: PLoS One. 2016 May 25;11(5):e0154504. doi: 10.1371/journal.pone.0154504 (PMC4880349; doi:10.1371/journal.pone.0154504)
Supplement: S3 Table — Region refers to which area of the body was covered by each ROI, sun refers to whether an individual ROI in a given series was directly or indirectly illuminated, and zebra (random factor) refers to the individual out of the 21 series that comprised the data set. Together, these variables accounted for 42% and 32% of the variance in d values in black stripes and white stripes, respectively. This and the analysis presented in S3 Table were performed using function ‘anovan’ in Matlab, using Type III sums of squares. (PDF) [file pone.0154504.s003.pdf]

	Black stripes					White stripes				
**Source**	**Sum Sq.**	**d.f.**	**Mean Sq.**	**F**	**Prob>F**	**Sum Sq.**	**d.f.**	**Mean Sq.**	**F**	**Prob>F**
**region**	0.19861	7	0.02837	1.98	0.0634	0.15662	7	0.02237	1.63	0.1331
**sun**	0.06295	1	0.06295	4.4	0.0382	0.00343	1	0.00343	0.25	0.6176
**zebra**	0.65751	20	0.03288	2.3	0.0033	0.51626	20	0.02581	1.89	0.0202
**Error**	1.58714	111	0.0143			1.5199	111	0.01369		
**Total**	2.72597	139				2.23436	139			

Table S2. ANOVA relating degree of polarization to region ilumination, region, and zebra. Region refers to which area of the body was covered by each ROI, sun refers to whether an individual ROI in a given series was directly or indirectly illuminated, and zebra (random factor) refers to the individual out of the 21 series that comprised the data set. Together, these variables accounted for 42% and 32% of the variance in *d* values in black stripes and white stripes, respectively. This and the analysis presented in Table S3 were performed using function 'anovan' in Matlab, using Type III sums of squares.
