# Supplementary material for: Zebras and Biting Flies: Quantitative Analysis of Reflected Light from Zebra Coats in Their Natural Habitat
Source: PLoS One. 2016 May 25;11(5):e0154504. doi: 10.1371/journal.pone.0154504 (PMC4880349; doi:10.1371/journal.pone.0154504)
Supplement: S4 Table — Sun azimuth was coded either east or west, because the data were taken in the tropics and the range of azimuths was therefore limited. Elevation was coded as low or high, with the criterion elevation being 45 degrees. The sun variable was as in Table 2, and orientation was a continuous variable, measured in degrees relative to the viewing direction, with 0 being head-right and 90 being head-away. Only illumination was individually significant, though many variables approached signficance. These independent variables accounted for 21% and 13% of the variance in black- and white-stripe d values, respectively. We interpret these results to mean that scene variables contributed to the differences between zebras, but that there was considerable residual variance that must be attributed to intrinsic differences between the reflectance properties of the pelts of different zebras. (PDF) [file pone.0154504.s004.pdf]

	Black stripes						White stripes				
**Source**	**Sum Sq.**	**d.f.**	**Mean Sq.**	**F**	**Prob>F**		**Sum Sq.**	**d.f.**	**Mean Sq.**	**F**	**Prob>F**
**azimuth**	0.01721	1	0.01721	1	0.3144		0.01891	1	0.01891	1.24	0.2667
**elevation**	0.06425	1	0.06425	3.8	0.0532		0.00004	1	0.00004	0	0.9609
**region**	0.21457	7	0.03065	1.8	0.0894		0.16062	7	0.02295	1.51	0.1693
**sun**	0.10048	1	0.10048	6	0.016		0.01489	1	0.01489	0.98	0.324
**orientation**	0.04103	1	0.04103	2.4	0.1214		0.05401	1	0.05401	3.56	0.0616
**Error**	2.15966	128	0.01687				1.94448	128	0.01519		
**Total**	2.72597	139					2.23436	139			

Table S3. ANCOVA relating degree of polarization to a set of scene variables that might be expected to contribute to the differences between zebras seen in the analysis reported in Table S2. Sun azimuth was coded either east or west, because the data were taken in the tropics and the range of azimuths was therefore limited. Elevation was coded as low or high, with the criterion elevation being 45 degrees. The sun variable was as in Table 2, and orientation was a continuous variable, measured in degrees relative to the viewing direction, with 0 being head-right and 90 being head-away. Only illumination was individually significant, though many variables approached significance. These independent variables accounted for 21% and 13% of the variance in black- and white-stripe *d* values, respectively. We interpret these results to mean that scene variables contributed to the differences between zebras, but that there was considerable residual variance that must be attributed to intrinsic differences between the reflectance properties of the pelts of different zebras.
